# Increased rates of large‐magnitude explosive eruptions in Japan in the late Neogene and Quaternary

**DOI:** 10.1002/2016GC006362

**Published:** 2016-07-01

**Authors:** S. H. Mahony, R. S. J. Sparks, L. M. Wallace, S. L. Engwell, E. M. Scourse, N. H. Barnard, J. Kandlbauer, S. K. Brown

**Affiliations:** ^1^ School of Earth SciencesUniversity of BristolBristolUK; ^2^ Institute for GeophysicsUniversity of Texas at AustinAustinTexasUSA; ^3^ British Geological SurveyEdinburghUK

**Keywords:** volcano, ocean drilling, Japan, tephra

## Abstract

Tephra layers in marine sediment cores from scientific ocean drilling largely record high‐magnitude silicic explosive eruptions in the Japan arc for up to the last 20 million years. Analysis of the thickness variation with distance of 180 tephra layers from a global data set suggests that the majority of the visible tephra layers used in this study are the products of caldera‐forming eruptions with magnitude (M) > 6, considering their distances at the respective drilling sites to their likely volcanic sources. Frequency of visible tephra layers in cores indicates a marked increase in rates of large magnitude explosive eruptions at ∼8 Ma, 6–4 Ma, and further increase after ∼2 Ma. These changes are attributed to major changes in tectonic plate interactions. Lower rates of large magnitude explosive volcanism in the Miocene are related to a strike‐slip‐dominated boundary (and temporary cessation or deceleration of subduction) between the Philippine Sea Plate and southwest Japan, combined with the possibility that much of the arc in northern Japan was submerged beneath sea level partly due to previous tectonic extension of northern Honshu related to formation of the Sea of Japan. Changes in plate motions and subduction dynamics during the ∼8 Ma to present period led to (1) increased arc‐normal subduction in southwest Japan (and resumption of arc volcanism) and (2) shift from extension to compression of the upper plate in northeast Japan, leading to uplift, crustal thickening and favorable conditions for accumulation of the large volumes of silicic magma needed for explosive caldera‐forming eruptions.

## Key Points

- Increase in volcanic activity in Japan at ∼8, 4–6, and ∼2 Ma
- Tephra layers in distal sediment cores represent magnitude 6 or greater eruptions
- Tectonic changes driving rates of explosive volcanism in Japan

## 1 Introduction

Visible volcanic tephra layers in marine sediment cores are a major resource to study the history of volcanism at both global and regional scales [*Ninkovich and Donn*, 1976; *Cambray and Cadet*, 1994]. In ocean drilling, this record can extend back millions to a few tens of millions of years. This record is much more complete than the terrestrial record which deteriorates rapidly back in time [e.g., *Brown et al*., 2014; *Kiyosugi et al*., 2015] due to erosion, weathering, and burial. However, the marine tephra layer record is itself subject to biases, such as selection of larger magnitude eruptions, which are more likely to be preserved as visible ash layers. In this paper, we investigate the completeness and utility of the marine tephra layer record for understanding the igneous and tectonic processes that control the rates of explosive volcanism using the Japan region as a case study. Japan is used as the case study area due to the high density of ocean drilling locations, the complex and dynamic tectonic regime, and high quality of land‐based tephra studies. We calibrate this record against data on tephra layer thickness as a function of distance from source to constrain the bias in terms of magnitude. Our study also enables us to demonstrate a marked Pliocene to Quaternary increase in the rates of large magnitude silicic caldera‐forming eruptions in Japan, which can be linked to major changes in plate tectonics.

## 2 Tephra Layers in Marine Sediments Around Japan

**Figure 1:**
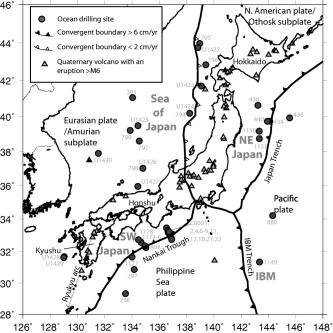
Map of study area, Japan. IODP/ODP/DSDP core locations (dark grey circles), Holocene volcanoes (light grey triangles), and the described regions, NE Japan, Sea of Japan, SW Japan, and the Izu‐Bonin arc (IBM). Drill sites are named in pale grey text.

We investigate tephra layers as records of explosive volcanism around Japan, identified from International Ocean Discovery Program (IODP; and previous ODP and DSDP programs) cores. Most drill sites that we used data from are typically several hundred kilometers from the nearest volcanic sources, allowing us to look at the very large magnitude volcanic events, which are able to deposit tephra further from source. Changes in rates of explosive eruptions are investigated in the overall data set, and also regionally: northeast Japan, Sea of Japan, and southwest Japan (Figure 1). Visible tephra layer preservation is a function of many parameters [*Sparks et al*., 1997; *Wetzel*, 2009; *Cassidy et al*., 2014; *Mahony et al*., 2014; *Jutzeler et al*., 2014; *Engwell et al*., 2014] including: location of source volcano; distance from source volcano; wind strength and direction; grain size distribution; atmosphere and ocean tephra transport processes; aggregation of fine ash particles; depositional environment, bioturbation, and coring processes/disturbances. Primary tephra fall layers can be difficult to distinguish from reworked tephra layers of secondary origin [*Manville et al*., 2009; *Cassidy et al*., 2014; *Mahony et al*., 2014]. The recorded tephra occurrences used in this study may have been formed by primary air fall, or may be secondary in nature, but both are of interest as they identify volcanic events. Volcanic records in deep‐sea cores may be influenced by plate movements [*Ninkovich and Donn*, 1976] due to temporal changes in proximity of the core location relative to volcanic sources. Below we demonstrate that visible tephra layers in deep‐sea cores that are distal (more than ∼100 km) from a volcanic arc are the consequence of large magnitude (M > 6) caldera‐forming explosive eruptions. The ocean drilling cores around Japan used in this study are typically hundreds to as much as 2000 km distance from possible source volcanoes (Figure 1).

**Figure 2:**
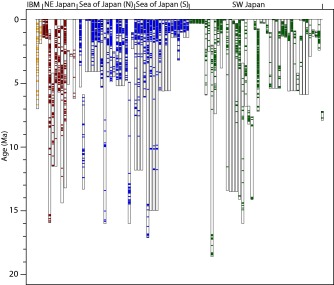
Representation of drill holes from the ocean regions around Japan. Colored zones represent at least one ash layer per ∼60 ka interval. Upper and lower limits of the drill hole are determined in the same manner as for the overall data analysis. The upper bounds are defined by the drilling records. Where appropriate the coring recovery is shown to start deeper than 0 m/0 Ma. The lower bounds of each drill hole are specified by the range of the age‐depth model used, the oldest tie point in the age‐depth model marks the deepest point in the hole for this study. Some drill holes may exceed the deepest marked level, however, if that point exceeds the age‐depth model limits then we cannot be sure of the deepest age. Minor breaks in coring recovery are not specified.

Data collection methods are described in *Mahony et al*. [2014]. This study compiled the visible tephra layer record in 93 drill holes at 45 coring locations (sites) from 17 expeditions around Japan (Figures 1 and 2). The data were extracted from shipboard handwritten visual core descriptions (VCDs). Where the VCDs were not available or when we were advised not to use them (e.g., because the original VCDs had been later edited when they were digitized, to have consistent lithology definitions with other linked expeditions), we used the digital records. Four thousand nine hundred and thirty‐six tephras, which had associated age depth models, were collated. The reported tephra thicknesses ranged from 0.05 to 150 cm (N.B. 150 cm is the length of a core section, so is the maximum that can be recorded on the VCD form). We used the most up to date published age‐depth models (see supporting information 1) to calculate the age of each visible tephra occurrence, by linear interpolation between tie points of known ages at given depths. These tie points are generated by magnetostratigraphy, biostratigraphy, or other dating methods. Records of tephra layers around Japan extend back to ∼20 Ma in some cores, and half of the cores extend back to >5 Ma (Figure 2). Cores from all three main regions extend back to >15 Ma. Even within a similar geographical area, tephra layers are present at different time intervals in different cores. Data are included from two sites (144‐880 and 185‐1149) that are closer to the Izu Bonin Mariana (IBM) arc than the Japan arc. However, the sites are distal (350–450 km) and the majority of tephra layers considered in the total Japan curve are found in locations far from the IBM, and so any localized IBM effect is negligible.

The record of visible tephra layers in ocean drilling cores is quantified for each core by the number of tephra layers per unit time interval. Numbers of tephra layers were initially counted per 100 ka for each drill hole. Next, the average numbers of tephra layers were calculated across all the drill holes with material to that age, for each 100 ka interval. These data were then averaged over 500 ka and 1 Ma intervals, and also for each region. These data are indicative of but do not represent the rate of volcanic events. Due to the averaging method being per drill hole with material of that age, we can include data from each individual drill hole rather than just using drill site composite records. This gives us a more complete record as there can be differences between drill holes just a few tens of meters apart.

There are several factors that control whether a particular volcanic event is recorded in a particular core. A tephra layer should ideally show great lateral consistency for a few hundred meters. However, a visible tephra layer may be present in one drill site and not in another, because of differences in tephra dispersal related to wind conditions and volcano location. Relatively small magnitude eruptions may form a visible tephra layer in cores close to the source but not in cores further away from the source. Visible tephra layers can be destroyed by bioturbation or erosion by bottom currents, with thinner layers being more prone to being destroyed. Sedimentation rate and paleoenviromental conditions can affect the rate of bioturbation, and so the preservation potential for tephra layers [*Wetzel*, 2009]. Here the tephra may still be present but is now cryptotephra, which is harder to recognize. Disturbances to core material during the coring processes can have a significant impact on tephra occurrence. Core material can be destroyed, extended so that it no longer represents the primary thickness, or reorganized in terms of grading and stratification [*Jutzeler et al*., 2014]. These more localized processes are indicated by variability in visible ash layers observed in the same time interval in closely spaced cores.

**Figure 3:**
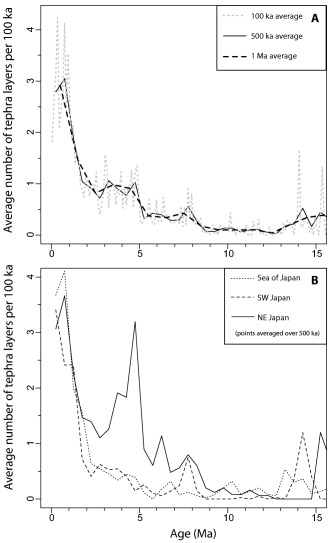
Average number of tephra occurrences with time. Numbers of tephra layers were initially counted per 100 ka for each drill hole. Average numbers of tephra layers were calculated across all the drill holes with material to that age, for each 100 ka interval. These data were then averaged over 500 ka and 1 Ma intervals, and also for each region. (a) All the data averaged over 100 ka (grey dotted line), 500 ka (solid black line), and 1 Ma (black dashed line) intervals. (b) Data divided by region, using a 500 ka average. Curves represent: northeast Japan (solid line), southwest Japan (dashed line), and the Sea of Japan (dotted line).

Here we choose to represent these data as a proxy for the volcanic event rates by averaging the number of tephra layers per unit time for a collection of cores (Figure 3). We have chosen two scales for averaging, namely the whole Japan region and a small number of subregions (such as core in the Sea of Japan). In each subregion, the numbers of tephra layers per unit time interval vary from one drill hole to another (Figure 2). Variation can be attributed to missing data due to absence of core material or preservation issues and additionally the same layer could occur in different time intervals due to the uncertainty in age models. Thus, the event rates are inferred to be lower bounds on the actual event rate. Below we compare these minimum event rates to event rates determined independently, from the terrestrial geological record.

We have not calculated the variance of the number of tephra layers as it can be inappropriate to make an estimate for nonnormal distributions and problematic too for data which include zeros [e.g., *Reimann and Filzmoser*, 2000]. Although there are standard methods for transforming data with zeros to enable variance to be calculated [e.g., *McDonald*, 2014], the choice of method depends on understanding the meaning of the zero values. In our case, there could be different reasons as discussed above for zero values, so a full understanding is lacking.

Peaks in explosive volcanism in the mid (15–13 Ma) Miocene are based on only a few cores and require additional cores to confirm. Rates of large magnitude explosive volcanism using visible tephra layers as a proxy are low 13–8 Ma then increase from 8 Ma, and again at 2.5–2 Ma (Figure 3a). Regional trends (Figure 3b) suggest the Sea of Japan and southwest Japan record generally low rates of large magnitude explosive eruptions until 5–6 Ma, with a peak in southwest Japan ∼8 Ma. Northeast Japan records high rates from 8–3.5 Ma, with a peak at 5 Ma. Further marked increases in explosive volcanism occurred between 1–2 Ma (Figure 3a), dominantly recorded in southwest Japan and southern Sea of Japan regions (Figure 3b). Finally, a pertinent observation is that many tephra layers in ocean drilling cores, which are at distal locations (e.g., mostly much greater than 100 km from the nearest volcano), consist predominantly of rhyolitic glass particles, indicating an association with silicic volcanism.

## 3 Magnitudes of Eruptions Recorded as Visible Tephra Layers

We adopted an empirical approach to constrain the likely magnitudes of the visible tephra layers in the deep‐sea core record. Visible tephra layer preservation is a function of eruption magnitude, with thickness generally increasing as magnitude increases at any given distance within the depositional footprint of the tephra. Thinner layers are more easily removed from the visible sediment record by mixing with sediment (forming cryptotephra) or eroded [*Wetzel*, 2009].

We calibrated the magnitudes (M) of the eruptions that form visible tephra layers in Japanese ocean drilling cores by analysis of a global data set of 181 isopach maps of Quaternary tephra fall deposits from large‐magnitude explosive eruptions collated in the LaMEVE database (Version 1, accessed October 2013) [*Crosweller et al*., 2012; *Brown et al*., 2014]. We extracted the actual thickness measurements used to construct each isopach map to create a database consisting of the event (deposit) name, reference, tephra thickness (cm), distance from source (km), and magnitude of each eruption analyzed (see supporting information 2). Coordinates were extracted for each measurement with the map project function in GMT [*Wessel and Smith*, 1991] or the published scale was used to measure distance from source using ImageJ. In cases of multiple isopach maps for different units of a single eruption, thickness from the most voluminous phase was considered.

Table 1 presents the number of isopach maps and the number of associated thickness measurements in our data set in magnitude bins. Frequency of different magnitude (M) eruptions decreases strongly as a function of magnitude [*Deligne et al*., 2010], following a power law up to about M7. Table 1 also shows the expected numbers of each eruption magnitude normalized to the number of M6 < 6.9 events that would be expected from the event frequency distribution [from *Deligne et al*., 2010]. The difference between the observed and expected numbers for M > 4 to M < 6 data is a consequence of recording biases. M > 4 to M < 6 data that inform the magnitude‐frequency relationship in *Deligne et al*. [2010] are derived largely from historic reporting, while M > 6 data are mostly from the geological record. Isopach maps of M > 6 eruptions are better recorded than M > 4 < 4.9 and M > 5 < 5.9. While a large number of data sets were collected for eruptions of M < 6.9, few published data sets are available for eruptions of M > 7 and data on M > 8 events exceeds that for M7. Thus, in relative terms, M < 6 deposits are underrepresented in our data set, and M > 8 are overrepresented. These data biases need to be taken into consideration in interpretation.

**Table 1:** Number of Isopach Maps and Thickness Measurements Collected From All Around the World for Each Magnitude Range Bin^a^

Magnitude	Number of Isopach Maps	Number of Thickness Measurements
8 ≤ 8.9	9	298
7 ≤ 7.9	6 (5)	88
6 ≤ 6.9	43 (43)	2097
5 ≤ 5.9	72 (495)	2453
4 ≤ 4.9	50 (2280)	1790
Total	181	6796

a Magnitude data are linked to events in the LaMEVE database [*Crosweller et al*., 2012]. The numbers in parentheses are the expected number of events normalized to the number of events in the magnitude 6 ≤ 6.9 bin if the distribution was to follow the global magnitude frequency relationship reported in *Deligne et al*. [2010, Table 5]. There is only data for eruptions where the thickness or isopach data are available, and therefore, the number of isopachs is always going to be much less than the actual number of eruptions.

**Figure 4:**
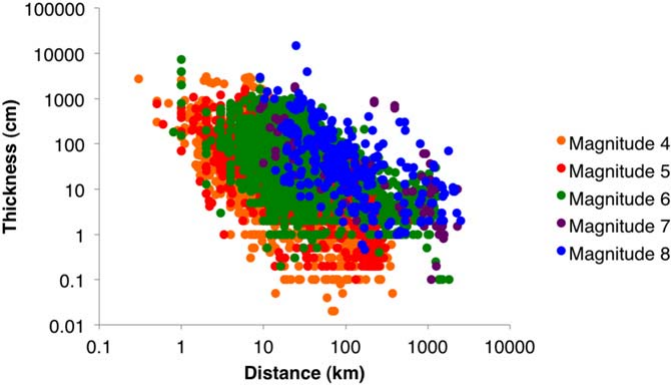
Tephra thickness as a function of distance from source for each magnitude interval.

Figure 4 shows a clear relationship between thickness and distance from source for each magnitude interval. These data were analyzed to assess the probability of observing a deposit exceeding a given thickness at a given distance from the source volcano. The data show a large spread in thickness measurements for each magnitude and distance, with the larger magnitude eruptions having greater thickness at greater distances from the source. Azimuth of a given deposit from source was not taken into account and likely contributes to the large range in thickness at a given distance for each magnitude through the effects of meteorological conditions.

For each magnitude and 50 km distance bin, the number of events was calculated with thickness values recorded either at that distance or greater (Table 2). Our analysis assumed that if a thickness measurement of an event is not recorded for that distance bin interval but is recorded at some greater distance then the event is also included in the count for that particular distance bin interval. The percentage number of events preserved as a deposit to a given distance and magnitude was then calculated using the total number of measurements for the distance of interest for all magnitudes (Table 2).

**Table 2:** Number of Events for Each Distance Bin, Increasing in 50 km Intervals and Magnitude, and Expressed as a Percentage Relative to the Total Number of Events for Each Distance Bin in Parentheses

Distance (km)	Magnitude					
	4	5	6	7	8	Total
>50	50 (27.8)	72 (40)	43 (23.9)	6 (3.3)	9 (5)	180
>100	11 (14.3)	26 (33.8)	26 (33.8)	5 (6.5)	9 (11.7)	77
>150	8 (14.5)	16 (29.1)	18 (32.7)	4 (7.3)	9 (16.4)	55
>200	4 (9.8)	10 (24.4)	15 (36.6)	3 (7.3)	9 (22)	41
>250	2 (5.7)	8 (22.9)	15 (42.9)	2 (5.7)	8 (22.9)	35
>300	2 (6.1)	7 (21.2)	15 (45.5)	2 (6.1)	7 (21.2)	33
>350	2 (7.7)	6 (23.1)	12 (46.2)	2 (7.7)	4 (15.4)	26
>400	2 (9.5)	2 (9.5)	11 (52.4)	2 (9.5)	4 (19)	21
>450	0 (0)	2 (11.8)	10 (58.8)	1 (5.9)	4 (23.5)	17
>500	0 (0)	2 (11.8)	10 (58.8)	1 (5.9)	4 (23.5)	17

**Figure 5:**
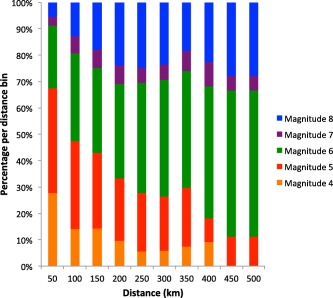
The number of events for each magnitude with data in a given distance bin expressed as a percentage relative to total number of events that distance bin.

Figure 5 shows the proportion of measurements in our data set that are attributed to a given magnitude interval as a function of distance. This plot gives a semiquantitative indication of probability of observing a tephra deposit from a particular magnitude interval at a given distance from source. However, there are clear biases in these proportions, including the paucity of data from isopach maps for M7–7.9. The probability of a given deposit being from a M4–4.9 or M5–5.9 eruption decreases with distance from source. The probability that a given deposit is from an M > 7 eruption increases with distance from source. At distances greater than 100 km from source, there are more M > 6 eruptions in each distance bin than smaller magnitude categories. For distances of 150–500 km, deposits from M > 6 eruptions dominate the geological record of preserved tephra layers. The ocean drilling cores around Japan are typically hundreds to as much as 2000 km distance from possible source volcanoes (Figure 1). We thus believe that most visible tephra layers are from very large magnitude eruptions (M > 6). Eruptions of M > 6 in the LaMEVE database are mostly associated with formation of calderas and so a further inference is that the visible tephra layers in ocean drilling cores is a record of caldera‐forming eruptions. We now refine this analysis to infer likely magnitudes of the visible tephra layers by comparison of the event rates from the cores with event rates constrained by the terrestrial record of tephras and calderas.

## 4 Comparison of Terrestrial Volcanic Versus Ocean Tephra Records

There is an excellent record of explosive volcanism on Japan in the form of detailed tephrochronology and records of caldera‐forming events. Here we compare land‐derived records with the ocean drilling data on visible tephra layers.

### 4.1 Detailed Comparison Of Ocean Drilling And Land‐Derived Volcanic Event Rates Over The Last 100 ka

Land‐based records of the Quaternary volcanic history of Japan were collected from the LaMEVE database and the Collapse Caldera DataBase (CCDB) [*Geyer and Marti*, 2008] for eruptions of magnitude 5 or greater. Records from the last 100 ka showed the best preservation of land deposits, so volcanic events of varying magnitude were compared with the relevant deep‐sea records. The record in the oceans for the last 100 ka will also not be affected by changes in plate tectonics or plate movements. Table 3 presents the number of events from this land‐derived terrestrial record for magnitude intervals of 5–5.9, 6–6.9, and 7–7.9, in 10 ka intervals. The data for M5–5.9 are likely affected by increased under recording going back in time [*Kiyosugi et al*., 2015]. Overall, the average rate of visible ash layers in the ocean drilling cores is 3.65 per 100 ka (Table 3), while the rates from the terrestrial record for varying magnitudes are (in parentheses): M5–5.9 (188), M6–6.9 (50), and M7–7.9 (10). For reasons already discussed, the average number of tephra layers per time interval in the ocean drilling cores is a minimum measure of event rate. In Table 3, we also report the maximum number of tephra layers in any individual core for each 10 ka time interval. In this case, each maximum value is a real event. Summing these maximum values gives 37 events in the last 100 ka, a value quite close to the 50 events M > 6 recorded in the terrestrial record. Thus, the comparison supports our inference from an analysis of global tephra thicknesses that the ocean drilling record is one of eruptions with magnitudes M > 6.

**Table 3:** Volcanic Event Rates Per 10 ka Interval, Over the Last 100 ka^a^

Age Interval (Ma)	No. of M5–5.9 Eruptions	No. of M6–6.9 Eruptions	No. of M7–7.9 Eruptions	Average Number of Tephra Layers in Cores	Maximum Number of Tephra Layers in One Drill Hole
0–0.01	35	3	1	0.41	4
0.01–0.02	19	5	1	0.27	2
0.02–0.03	24	4	1	0.45	2
0.03–0.04	21	7	2	0.52	6
0.04–0.05	13	5	1	0.39	4
0.05–0.06	17	4	0	0.42	4
0.06–0.07	13	5	1	0.18	2
0.07–0.08	13	2	0	0.24	2
0.08–0.09	19	9	2	0.44	9
0.09–0.1	14	6	1	0.33	2
Total/event rate per 100 ka	188	50	10	3.65	37

a The numbers of M5, M6, and M7 eruptions are from the LaMEVE database and the Collapse Caldera DataBase (CCDB) [*Geyer and Marti*, 2008]. The average number of tephra layers is averaged across all drill holes with material of that age. The maximum number of tephra layers per hole is the maximum number in any one drill hole with material of that age. The bottom “Total” row is the sum of each of the M5, M6, M7 columns to give the event rates over the total 100 ka period. The “Total” row for the average number of tephra layers in cores is the mean of all of the 10 ka intervals in that column multiplied by 10, to take it from the 10 ka to the 100 ka average rate. The “Total” row for the maximum number of tephra layers in one drill hole is the sum of the values in that column from 0 to 100 ka, to give the 100 ka maximum event rate.

### 4.2 Holocene to Pliocene Comparison of Ocean and Land‐Based Records, Showing Trends Through Time and Volcanic Event Rates

Tephrochronology of land and lake deposits in Japan extend back to the Pliocene [*Kimura et al*., 2015; *Yoshida et al*., 2013]. Many tephra horizons have been correlated with different regions of Japan and even with particular volcanoes through geochemical fingerprinting. With nearly 5000 tephra layers recorded in the ocean drilling cores around Japan, there are almost no comparable geochemical data to identify sources in the marine record, although a small number of very young tephra layers have been correlated to their sources (e.g., Aso) [*Machida*, 2002]. *Kimura et al*.'s [2015] terrestrial tephra record demonstrates a hiatus in volcanic activity in northeast Japan from 3.5–1.5 Ma, which is when we also observe a lower numbers of tephra layers in the ocean drilling cores. The peak in marine tephras in northeast Japan at ∼5 Ma (Figure 3b) is not seen in the *Kimura et al*. [2015] record, but their northeast Japan record does not extend much past 4 Ma. The terrestrial tephra data [*Kimura et al*., 2015] for Kyushu, southwest Japan and central Japan sources can be used to do a regional comparison with the ocean drilling data for southwest Japan (Figure 1). Our records for southwest Japan and Sea of Japan are fairly similar over the 4–0 Ma period, with lower levels of volcanic activity until around 2.5 Ma, then a sharp increase in activity until recent times (Figure 3b). *Kimura et al*. [2015] show a terrestrial central Japan pulse of activity from 2.5–1.8 Ma and that the Kyushu terrestrial record increases from 1 Ma to present. When the records of widespread terrestrial tephras for these regions are combined (Table 4), we see the same pattern as in the ocean drilling data, i.e., a fairly continuous increase from 2.5 Ma to present, but the terrestrial record shows that the increase is also associated with shifts in the location of the volcanoes. Table 4 shows data in 0.5 Ma intervals back to 5 Ma to compare the terrestrial tephrochronology record with the marine record. The number of widespread terrestrial tephras from 0–0.5 Ma (25; Table 4) lies within the range defined by the average number of tephra layers in ocean cores (2.79; Table 4), and the maximum number of tephra layers in any one drill hole (88; Table 4). In fact, this is true of each half million year interval back to 5 Ma (Table 4). The maximum from any individual drill hole from 0–0.5 Ma is located in southwest Japan (88), with other regional maximums of 69 and 56 recorded in the Sea of Japan and northeast Japan (Table 4). Taken in half million year intervals back to 5 Ma, the numbers of widespread terrestrial tephras decreases markedly in intervals after the 0–0.5 Ma, which we attribute as an artefact of poor preservation [*Kiyosugi et al*., 2015]. In contrast, the ocean tephra records indicate a marked increase in explosive volcanism at about 2 Ma. These data imply that the marine record is more complete.

**Table 4:** Comparison of Ocean Drilling Tephra Records With Land‐Derived Widespread Terrestrial Tephra Data From *Kimura et al*. [2015]^a^

Time Interval (Ma)	Total Number of Widespread Terrestrial Tephras	Average Tephra From Ocean Cores	Maximum No. Tephra in One Drill Hole From All Ocean Cores	Maximum Tephra in One Drill Hole in Sea of Japan	Maximum Tephra in One Drill Hole in SW Japan	Maximum Tephra in One Drill Hole in NE Japan
0–0.5	25	2.79	88	69	88	56
0.5–1	9	3.05	52	52	47	48
1–1.5	8	1.89	84	25	84	32
1.5–2	8	1.04	22	22	22	22
2–2.5	5	0.91	14	12	14	14
2.5–3	5	0.72	22	13	22	14
3–3.5	1	1.06	24	14	24	14
3.5–4	6	0.90	23	14	18	23
4–4.5	3	0.77	27	8	17	27
4.5–5	1	1.03	56	8	9	56
Total (0–5 Ma)	71	1.42	214	128	157	214

a The average tephra per 500 ka from ocean cores is averaged across all the data. The “total” for this column is the average of the values above. The columns for maximum numbers of tephra are the maximum number of tephra layers in any one drill hole in that time interval and within any spatial constraints. The “total” row for the maximum ocean tephra layer columns is not the sum of the 500 ka intervals above, but is the maximum number of tephra layers from 0 to 5 Ma in any one drill hole. The sum of the above rows would give a combination of maximum values for individual 500 ka intervals from many different drill holes.

Over the last 5 Ma, *Kimura et al*. [2015] record 71 widespread tephras around Japan, *Yoshida et al*. [2013] describe 81 calderas in northeast Japan, and we record a maximum of 214 tephras in a single drill hole during this 5 Ma time interval. We attribute the greater number of marine events to several factors. First our global tephra magnitude‐thickness data (Figure 5) suggest about 20–30% of the marine tephra events are M < 6 (Figure 5). However, most of these marine tephra events are M > 6 (Figure 5) and therefore likely associated with caldera formation. Thus the difference between the numbers reflects the fact that there are many more Pliocene‐Quaternary calderas in Japan in areas other than those reported by *Yoshida et al*. [2013] in the northeast. Additionally, calderas are commonly associated with more than one large magnitude explosive eruption, for example with the Aso caldera, which has produced four major tephra layers [*Machida*, 2002]. Finally, as already discussed in relation to the data in Table 4, the marine record is more complete than the terrestrial record.

## 5 Changes in Visible Ash Layer Occurrence

Our observations (Figures 2 and 3) of visible tephra layers show significant changes in space and time. These changes could be related to several factors: plate motions, tectonic or melt generation processes, location of volcanic sources, environment of volcanism, and prevailing winds. Cores located on subducting plates are expected to preserve increasing numbers of tephra layers as they approach an active volcanic arc [*Ninkovich and Donn*, 1976] and more tephra layers might be expected in the dominant downwind direction away from an active arc. Tectonic setting and plate boundary dynamics affect volcanism, e.g., strike‐slip plate boundaries and shallow flat slab subduction typically generate little or no arc volcanism. Tectonic and igneous processes in the subducting plate and arc crust may affect rates of explosive volcanism; for example, compressive tectonics along with thickening crust may favor generation and accumulation of large silicic magma bodies. Eruptive environment (submarine versus subaerial) has influence, with subaerial volcanoes more likely to produce more explosive volcanism, thus more ash [*Fiske et al*., 1998; *Carey et al*., 2014].

We now consider these different factors in turn, starting with those that we infer are less important.

### 5.1 Wind

**Figure 6:**
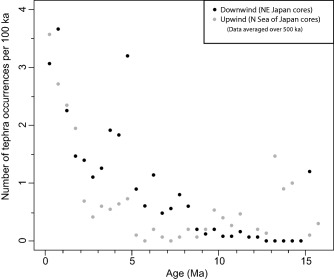
Average numbers of tephra occurrence upwind and downwind of volcanic sources in northern Japan. Upwind sources (grey circles) are located in the northern part of the Sea of Japan. Downwind sources (black circles) are in NE Japan. The data are similar, suggesting that prevailing wind direction has minimal effects in this region.

In Japan, dominant westerly stratospheric winds influence the deposition of tephra from small to intermediate magnitude eruptions. However, M > 6 caldera‐forming eruptions discharge tephra to heights of tens of kilometers into the atmosphere [*Sparks et al*., 1997], forming powerful giant umbrella clouds. For M > 6.5, umbrella clouds can spread ash hundreds of kilometers in the upwind and cross‐wind directions [*Baines and Sparks*, 2005]. Wide dispersal of tephra layers in Japan is well documented; for example, tephra layers of M > 7 from the Aso caldera in Kyushu are found in Hokkaido [*Machida and Arai*, 2003]. Thus dispersal of tephra layers with M > 6 is not as strongly affected by wind direction [*Suto et al*., 2007] as smaller magnitude eruptions. Tephra layer occurrence in cores from the northern Sea of Japan, upwind of volcanic sources, are similar to rates on the downwind side (NE Japan) as shown in Figure 6. This observation supports the inference that wind direction is not a major factor in dispersal of tephra from large magnitude (M > 6) Japanese eruptions.

### 5.2 Plate Movements

Late Cenozoic plate movements would not have affected the tephra layer record at the sites on the upper plate in Japan, due to relatively small horizontal tectonic motions of the core sites relative to volcanic sources. Sea of Japan opening occurred between 30 and 14 Ma [*Sato and Amano*, 1991] although the exact timing is disputed [*Otofuji et al*., 1991; *Tamaki et al*., 1992]. The Sea of Japan core locations relative to potential arc volcanic sources changed very little during the last 20 Ma.

**Figure 7:**
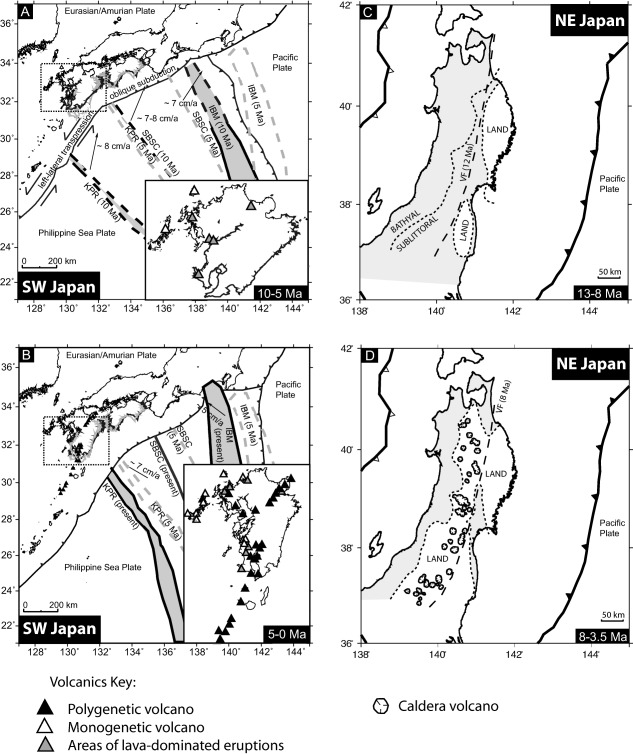
Tectonic and volcanic reconstructions of (a, b) southwest and (c, d) northeast Japan, in time slices since the Miocene. In Figure 7b, calderas are not marked, but occur along the NE‐SW trending polygenetic volcano arc. Figures 7a and 7b after *Mahony et al*. [2011] and Figures 7c and 7d modified from *Sato* [1994].

**Figure 8:**
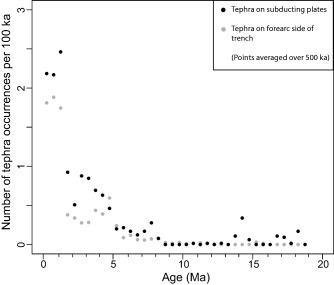
Plate versus fore‐arc tephra layer numbers. Tephra layers on subducting plates (black filled symbols) versus fore‐arc side of the trench (grey filled symbols). The combined data set has been divided into tephra located on the down‐going Pacific and Philippine Sea Plates versus tephra located in the fore‐arc/wedge side. The plate‐side tephra does not show an increase with time when compared with the wedge‐side data.

There is obviously larger motion of core sites on the subducting plate relative to the volcanic sources over the time frame of our study. Fifteen southwest Japan sites are on the overriding plate, while eleven are on the subducting plate. During 18–7 Ma, the locations of the southwest Japan subducting plate cores would have moved northeast relative to the plate boundary at ∼ 5–8 cm/yr (∼50–80 km/Myr) [*Hall*, 1998; *Mahony et al*., 2011; *Pickering et al*., 2013] (Figure 7), then moved directly towards the plate boundary at ∼5–6 cm/yr since ∼7 Ma. In northeast Japan, five sites are on the overriding plate, and one is on the incoming Pacific Plate. The Pacific Plate site has been moving toward northeast Honshu at 5–8 cm/yr since ∼20 Ma [*Regalla et al*., 2013; *Doubrovine and Tarduno*, 2008]. Cores located on subducting plates might be expected to preserve increasingly larger numbers of tephra layers as they approach an active volcanic arc [*Ninkovich and Donn*, 1976]. However, for subducting plate‐side, numbers of tephra layers do not increase more rapidly toward the present than those on the fore‐arc side (Figure 8). Although the effect of plate movements must be real for those sites that have approached the arc, they are not discernible in the data and we conclude that plate movement affecting the distance from the source is not a major factor in controlling the overall patterns.

### 5.3 Tectonic Controls on Volcanism

Our analysis indicates a marked increase in rates of large magnitude explosive volcanism since ∼8 Ma. Major tectonic changes have occurred in Japan since 20 Ma. The plate boundary in southwest Japan was predominantly sinistral strike slip with a minor convergent component between 18–7 Ma [*Mahony et al*., 2011; *Pickering et al*., 2013], then a change in Philippine Sea Plate kinematics led to arc‐normal Philippine Sea Plate subduction ∼6 Ma [*Hall et al*., 1995; *Mahony et al*., 2011]. Changes in Philippine Sea Plate motion are likely driven by forces on its boundary with the Pacific Plate. The change from Philippine Sea Plate strike‐slip motion at 6–8 Ma is attributed to changes in slab pull forces acting at the Pacific Plate subduction boundary [e.g., *Faccenna et al*., 2012].

Prior to 8 Ma, relative motion between the Philippine Sea Plate and southwest Japan was dominated by strike‐slip faulting (and perhaps some slow convergence) and was associated with low rates of back arc volcanism [*Kamata and Kodama*, 1994; *Mahony et al*., 2011]. Between 13 and 8 Ma subduction of the Pacific Plate was associated with early transition from Sea of Japan opening to a contractional tectonic regime, and most arc volcanism in northeast Japan was likely submarine [*Sato*, 1994]. A submarine environment may have inhibited production of widespread ash dispersal by explosive volcanism. Moreover, a relatively thin arc crust that was stretched during the opening phase of the Sea of Japan did not favor generation and accumulation of large bodies of silicic magma. Taken together, the overall tectonic environment in both northeast and southwest Japan prior to ∼8 Ma did not favor large magnitude explosive volcanism.

We attribute the increase in rates of caldera‐forming explosive eruptions from 8 Ma in Japan to interacting tectonic and igneous processes. On the large scale, the onset of increasing explosive volcanism is attributed to both uplift and eventual exposure of NE Japan, as well as changes in plate boundary dynamics in both southwest and northeast Japan, which affected magma production and favored the generation of large volumes of silicic magma necessary for large caldera‐forming eruptions.

Southern Kyushu has rotated 30° anticlockwise since 6 Ma, accompanied by back‐arc extension and more rapid subduction [*Kodama et al*., 1995]. Following extension and subsidence during Sea of Japan opening between 30–14 Ma, northeast Japan underwent a transition to uplift and eventual contraction [*Sato*, 1994; *Regalla et al*., 2013] from ∼13 Ma to present. Around 8 Ma, much of the western half of northern Honshu became uplifted above sea level [*Sato*, 1994], and the early stage of a caldera‐dominated arc formed, with northeast‐southwest compression [*Yoshida et al*., 2013]. A later stage of caldera‐dominated arc has been identified in the terrestrial record in northeast Japan from 5.3–3.5 Ma [*Yoshida et al*., 2013], concurrent with the large increase in explosive volcanic activity seen in our data (Figure 3b).

Between 8 and 5 Ma major changes occurred. Subduction of the Philippine Sea Plate became more arc normal [*Seno and Maruyama*, 1984; *Seno*, 1989; *Hall*, 1998] and a marked increase in overall volcanism occurred in Kyushu [*Mahony et al*., 2011; *Pickering et al*., 2013]. The peak in northeast Japan volcanism, beginning around 5 Ma, is concurrent with the change in absolute direction of motion of the Pacific Plate which was associated with a larger component of compression normal to the Japan trench [*Pollitz*, 1986; *Sato*, 1994; *Regalla et al*., 2013]. Formation of silicic volcanism and calderas in northern Honshu can be attributed to an “intermediate stress regime” between 8–3.5 Ma [*Sato*, 1994], with horizontal and vertical stress fields being nearly equal. Uplift of the region also initiated at this time and likely was associated with magmatic underplating [*Sato*, 1994] and a change in slab dynamics [*Regalla et al*., 2013]. The regional switch in arc crust conditions from extension to compression favored silicic magma generation in the crust and accumulation of large magma bodies.

A further marked increase in large magnitude explosive volcanism is indicated in the marine tephra record around 2–1.5 Ma (Table 4). This increase in explosive volcanism is dominated by southwest Japan and southern Sea of Japan data. Data from the northern part of the Sea of Japan also show a less pronounced increase in average numbers of tephra layers from 1.5 Ma. The increased volcanism during the Quaternary is also noted in other circum‐pacific regions, e.g., the Kamchatka‐Kurile and Aleutian arcs [*Prueher and Rea*, 2001]. At 2 Ma, the Philippine Sea Plate changed subduction direction from NNW to NW, leading to a shift to dextral motion on the Median Tectonic Line [*Kamata and Kodama*, 1994] in southwest Japan, and the change to southwesterly migration of the subduction point of the Kyushu Palau ridge [*Mahony et al*., 2011]. The large and still active Kyushu‐Ryukyu arc calderas were formed in this period. Enhanced mantle magma production rates could also be associated with an increase in subduction rate linked to increased rates of back arc extension, slab roll‐back and steepening of the subduction angle allowing greater inflow of melt‐producing asthenosphere related to corner flow. Marked increase in mantle magma flux could then lead to rapid silicic magma generation. In northern Honshu, from 2.4 Ma to present, shortening in the Sea of Japan uplifted the entire region, created a compressive environment and likely increased crustal thickness, promoting favorable conditions for formation of large volumes of silicic magma over the last 2–3 Myrs. The combination of tectonic regimes through time (compressional/trans‐tensional) primed the mantle under Japan for when uplift and slab roll‐back led to ideal conditions for silicic melt generation in the arc crust and caldera formation.

## 6 Conclusions

The large‐scale physical processes of tectonics and volcanism/magmatism are inextricably linked, and we demonstrate how changes in tectonics can drive changes in the rate of large magnitude volcanic events. Our data set enables a comprehensive study of the ocean drilling programs cores located around Japan. Our detailed eruption deposits study and comparison with global thickness data from 180 deposits, reveals that tephra layers observed in ocean drilling cores located at least 100 km from source volcanoes are likely to be M > 6. This inference is also supported by comparison of the marine tephra record with the Pliocene‐Quaternary terrestrial tephra record and observed numbers of calderas. We observe increases in numbers of volcanic tephra layers at 8, 4–6, and ∼2 Ma both in the data set as a whole, and also when divided by core location regions. The increase in numbers of volcanic tephra layers in northeast Japan starting at 8 Ma is broadly consistent with the increases in studies of the Pliocene‐Quaternary terrestrial record [*Yoshida et al*., 2013], but the marine record extends back further in time and is also shown to be more complete. Increases in activity seen on all regions of our study from ∼2 Ma to present, also fit well with the increases in rates of volcanic activity towards present noted in land‐based studies [*Kimura et al*., 2015]. Tectonic changes are the major driving force behind the late Cenozoic increases in rates of large magnitude explosive volcanism in Japan. These changes have led to conditions in which large bodies of silicic magma can accumulate within the arc crust, leading to favorable circumstances for explosive caldera‐forming eruptions.

## Supporting information

Supporting Information S1

Data Set S1

Data Set S2
