# Supplementary material for: Increased rates of large‐magnitude explosive eruptions in Japan in the late Neogene and Quaternary
Source: Geochem Geophys Geosyst. 2016 Jul 1;17(7):2467–79. doi: 10.1002/2016GC006362 (PMC5012118; doi:10.1002/2016GC006362)
Supplement: Supplementary file 1 — Supporting Information S1 [file GGGE-17-2467-s001.doc]

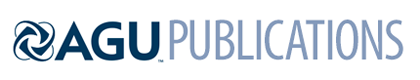


*Geochemistry, Geophysics, Geosystems*

Supporting Information for

**Increased rates of large magnitude explosive eruptions in Japan**

S.H. Mahony^1*^, R.S.J, Sparks^1^, L.M. Wallace^2^, S.L. Engwell^1,3^, E.M. Scourse^1^, N.H. Barnard^1^, J. Kandlbauer^1^ and S.K. Brown^1^

1. School of Earth Sciences, University of Bristol, Bristol BS8 1RJ, United Kingdom

2. Institute for Geophysics, University of Texas at Austin, Austin, TX 78758, USA

3. British Geological Survey, Murchison House, W Mains Rd, Edinburgh, EH9 3LA

*Email: Sue.Mahony@bristol.ac.uk

**Contents of this file**

**Additional Supporting Information (Files uploaded separately)**

Captions for Datasets S1 and S2

**Introduction**

This supporting information provides the raw data of the ocean drilling tephras and the thickness dataset.

Text S1.

Ocean drilling ash layer data. Core name follows the IODP naming protocol, e.g. 127-794A represents Expedition 127, Site 794, Hole A. Depth of ash is depth in metres to the bottom of the ash layer where known, either given in the VCD text or digital data file, or measured off the VCD diagram. Depth is measured in metres below sea floor (mbsf). Age of ash is the age in millions of years, calculated using the depth below sea floor and linear interpolation of points from appropriate age-depth models. The age-depth models used are listed in the other workbook tabs, with a link to the source of the age-depth model. Some are the age-depth models generated during the expedition, but where possible we have used post-cruise age-depth models, as they are likely to have improved or refined the original shipboard age-depth models.

Text S2.

Magnitude-distance from source tephra thickness data. Data taken from LaMEVE V.1, accessed October 2013.

Data Set S1.

Ocean drilling ash layer data. Core name follows the IODP naming protocol, e.g. 127-794A represents Expedition 127, Site 794, Hole A. Depth of ash is depth in metres to the bottom of the ash layer where known, either given in the VCD text or digital data file, or measured off the VCD diagram. Depth is measured in metres below sea floor (mbsf). Age of ash is the age in millions of years, calculated using the depth below sea floor and linear interpolation of points from appropriate age-depth models. The age-depth models used are listed in the other workbook tabs, with a link to the source of the age-depth model. Some are the age-depth models generated during the expedition, but where possible we have used post-cruise age-depth models, as they are likely to have improved or refined the original shipboard age-depth models.

Data Set S2.

Magnitude-distance from source tephra thickness data. Data taken from the LaMEVE database V.1, accessed October 2013. The event name, source volcano and magnitude refers to the records given in the LaMEVE database. The reference refers to the source of the isopach map for each eruption. Coordinates were extracted for each measurement with the mapproject function in GMT [Wessel and Smith, 1991] or the published scale was used to measure distance from source using ImageJ. In cases of multiple isopach maps for different units of a single eruption, thickness from the most voluminous phase was considered.
